# Breaking barriers: A triumph in pregnancy for a patient with Sjögren syndrome: A case report

**DOI:** 10.1097/MD.0000000000042326

**Published:** 2025-05-02

**Authors:** Kaiyue Hu, Shuxia Ma, Ruirui Li, Ying Zhou

**Affiliations:** a Department of Reproductive Medicine, Luoyang Maternal and Child Health Hospital, Luoyang, China; b Luoyang Branch of the National Center for Assisted Reproduction and Eugenics, Luoyang, China; c Key Laboratory of Reproduction and Genetics, Luoyang, China; d Institute of Reproductive Medicine, Luoyang, China.

**Keywords:** assisted reproductive technology, immunosuppressive agents treatment, infertility, pregnancy, Sjogren’s syndrome

## Abstract

**Rationale:**

Sjögren syndrome (SS) is a systemic autoimmune disease with a variety of clinical presentations. Successful pregnancies and deliveries among these patients remain challenging. This manuscript describes a case report of diagnosis, detailed clinical management, and successful pregnancy and delivery of a patient of SS, which treated by immunosuppressive agents, especially cyclosporine A (CsA) and assisted reproductive technology.

**Patient Concerns:**

A 31-year-old female had been experiencing persistent infertility with a 5-year-long history. However, the results indicated that patent fallopian tubes, an ovulatory cycle, and normal semen analysis.

**Diagnoses:**

After obtaining clinical manifestations, medical history, and the serum biochemical and salivary gland histopathology examination results, main causes such as tubal factor, impaired endometrial function, endocrine dysfunctions and male factors were excluded, and the final diagnosis was SS-induced autoimmune infertility.

**Interventions:**

The patient was treated with immunosuppressive agents and assisted reproductive technology. After 5 failed attempts of assisted reproductive technology treatment and treatment with 3 immunosuppressive agents, the application of a relatively potent immunosuppressive agent CsA was finally used and a high-quality frozen-thawed blastocyst embryo was transferred.

**Outcomes:**

A clinical pregnancy was confirmed. The newborn was delivered by elective cesarean section and showed normal development over the 1-year follow-up.

**Lessons:**

The accurate diagnosis of autoimmune infertility requires a combination of clinical presentation, medical history, and biochemical findings, and, if available, histopathology examinations. The role of immunosuppressive agents, especially CsA is crucial for the clinical management of autoimmune infertility.

## 1. Introduction

Infertility is defined as the failure to achieve pregnancy following 1 year of unprotected coitus.^[1,2]^ Globally, infertility is one of the most common medical conditions. Approximately 80 million people suffer from infertility.^[3–6]^ The most common causes of infertility include 3 major groups: female factors (30%), male factors (30%), and combined and/or unexplained factors (40%).^[7]^ Among female infertility causes, ovulation disorders account for 20%, tubal obstruction for 20%, and endometriosis for 10%. In addition, other causes include pelvic inflammatory disease and uterine problems.^[8,9]^ Male factors include defective spermatogenesis, defective transport, and ineffective delivery.^[10]^ Moreover, among the unexplained factors, growing evidence indicates the potential involvement of hyperhomocysteinemia,^[11–13]^ MTHFR gene polymorphisms,^[13–15]^ variations in folate pathway genes,^[13]^ vitamin B-complex deficiencies,^[13]^ hereditary thrombophilias,^[16,17]^ and autoimmune antibodies.^[18–21]^

Sjögren’s syndrome (SS) is a typical systemic autoimmune disorder characterized by lymphocyte infiltration of the exocrine glands. The characteristic blood markers include anti-SSA and anti-SSB antibodies. Over 80% of patients present with oral and ocular dryness, fatigue, pain, and other clinical manifestations.^[22,23]^ Up to date, only a few cases of SS have been reported in infertile female patients. To the best of our knowledge, this is the first report describing regimen selection, the dose adjustment and treatment duration in detail in infertile female SS patients with successful pregnancy and delivery.

## 2. Case presentation

In July 2020, a 31-year-old female educator with a 5-year long history of infertility presented to the Reproductive Medicine Department of our hospital. She appeared to be in good health without any physical abnormalities, with a weight of 59kg and a height of 162 cm. The couple got married in 2016. After marriage, the couple lived together, had no history of separation, and had coitus without contraception. Notably, in 2018, the couple underwent an initial fertility examination workup in our hospital. However, the results showed patent fallopian tubes, an ovulatory menstrual cycle, and a normal semen analysis. Nonetheless, the patient failed to conceive. The patient had normal pubertal development and reported spontaneous menarche at age 13 and regular menstrual cycles, with normal menstrual volume and no dysmenorrhea.

**Figure 1.**
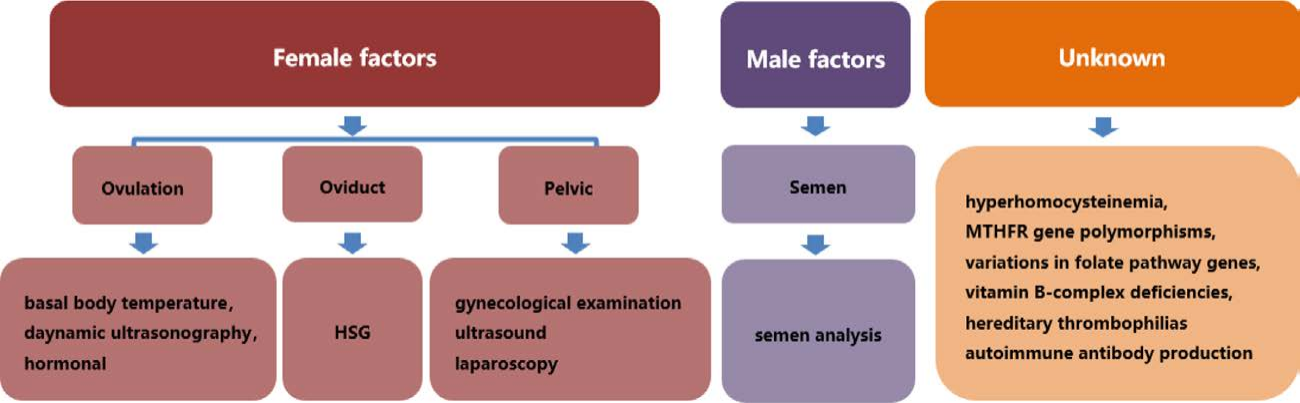
Classification chart of infertility factors and corresponding examination.

**Figure 2.**
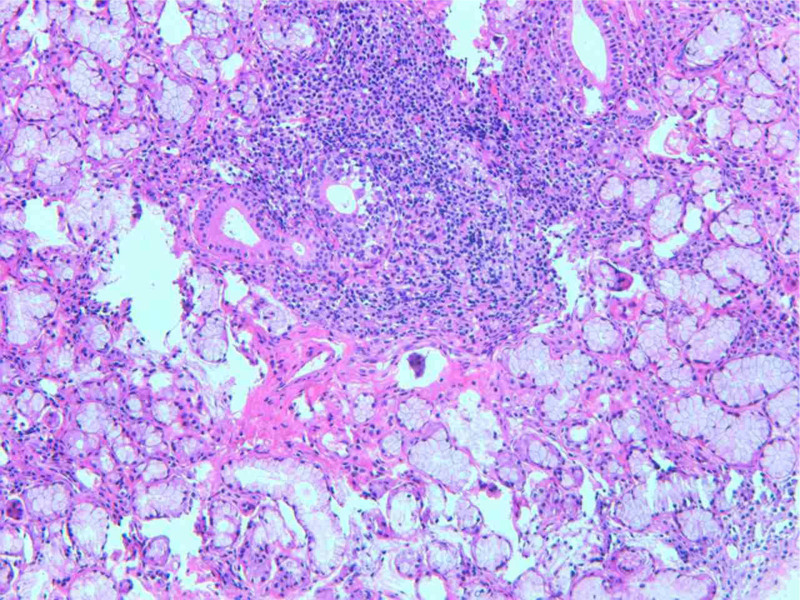
Pathological examination of the labial glands. The structure of the salivary gland lobules was present, and lymphocyte infiltration was found in the lobules and periductal stroma. Among them, 6 lesions with more than 50 lymphocytes were found.

**Figure 3.**
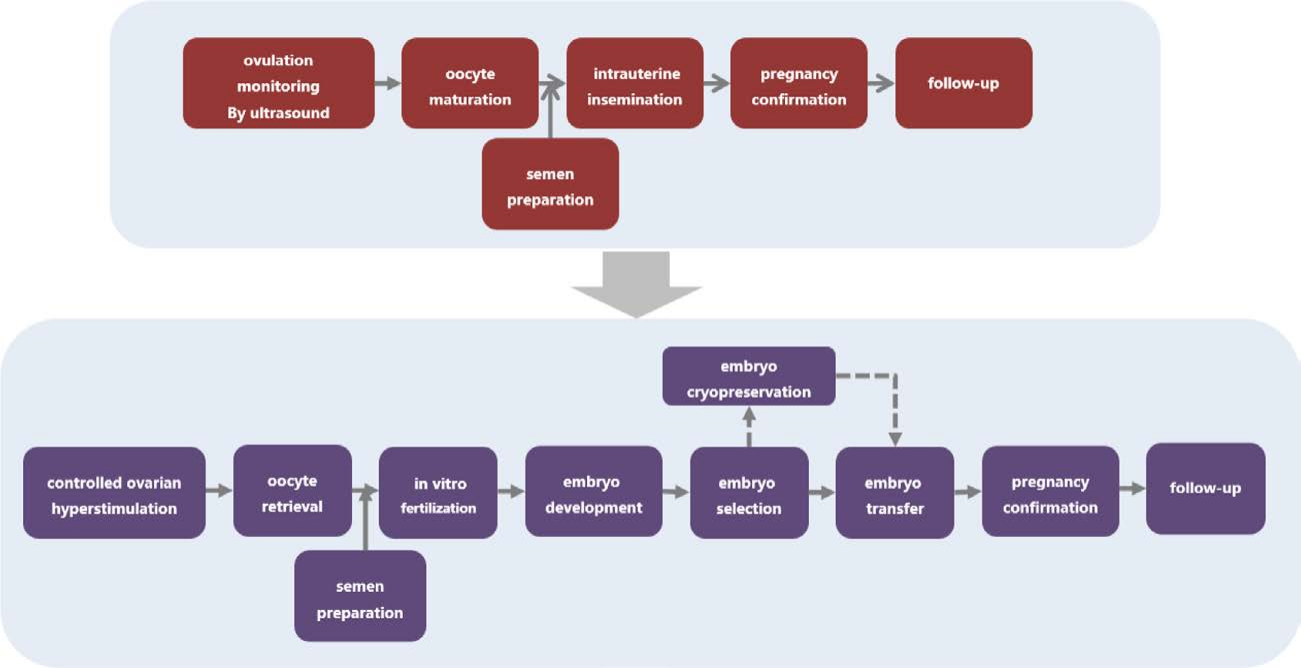
The workflow of ART. ART = assisted reproductive technology treatment.

Next, a comprehensive workup was performed to determine the possible causes of infertility in this couple. The screening process for infertility is shown in Figure 1. The only abnormalities in the screening results were immunological factors in the female patient, and the results of immunological examination were displayed in Table 1. Given the patient’s multiple positive autoantibodies, especially including 2 specific antibodies associated with SS (Table 1), therefore, the most likely diagnosis is SS. A pathological examination of salivary gland was then conducted to confirm the diagnosis, and the results revealed lymphocyte infiltration of the salivary gland lobules and periductal stroma. A total of 6 lesions with more than 50 lymphocytes were found (Fig. 2), consistent with the typical pathological feature in SS. Therefore, a definite diagnosis of SS was established based on the clinical features and histopathological findings. Next low-dose immunosuppressive agent therapy was resumed for the treatment of SS. The patient was prescribed hydroxychloroquine (HCQ) 100 mg BID orally since August 2020. In the meantime, assisted reproductive techniques (ART) were planned. Figure 3 illustrates a summary of the process of ART. In the regimen, in vitro fertilization (IVF) was performed to obtain oocytes and embryos, followed by 2 failed attempts of intrauterine insemination (IUI) treatment.

**Table 1:** The results of aPL and autoimmune antibodies.

Variable	Outcome	Associated diseases
aPL		
LAR	**−**	Antiphospholipid-antibody syndrome
aCL	**−**	
Anti-β2-GP1	**−**	
Autoantibody		
ANA	**+**	Connective tissue disease
Anti-dsDNA	**−**	Systemic lupus erythematosus
Anti-Jo-1	**±**	Polymyositis
Anti-PM-Scl	**−**	Connective tissue disease overlap syndrome
ARPA	**−**	Systemic lupus erythematosus
AMA-M2	**−**	Primary biliary cirrhosis
Anti-U1RNP	**−**	Mixed connective tissue disease
Anti-PCNA	**−**	Systemic lupus erythematosus
ANuA	**±**	Systemic lupus erythematosus
Anti-Sm	**−**	Systemic lupus erythematosus
Anti-SSA	**+**	Sjögren’s syndrome
Anti-SSB	**+**	Sjögren’s syndrome
Anti-ccp	**−**	Rheumatoid arthritis

aCL = anti-cardiolipin antibody, ANA = antinuclear antibodies, anti-ccp = anti-cyclic citrullinated peptide antibody, anti-dsDNA = anti-dsDNA antibody, anti-Jo-1 = anti-Jo-1 antibody, anti-PM-Scl = anti-PM-Scl antibody, ARPA = anti-ribosomal P antibody, AMA-M2 = anti-mitochondrial M2 antibody, anti-PCNA = anti proliferating cell nuclear antigen antibody, anti-Sm = anti-Sm antibody, anti-SSA = anti-SSA antibody, anti-SSB = anti-SSB antibody, anti-U1RNP = anti-ribonucleoprotein antibody, anti-β2-GP1 = anti beta-2-glycoprotein antibodies, ANuA = anti-nucleosome antibody, aPL = antiphospholipid antibodies, APS = antiphospholipid-antibody syndrome, CTD = connective tissue disease, LAR = lupus anticoagulant, MCTD = mixed connective tissue disease, PBC = primary biliary cirrhosis, RA = rheumatoid arthritis, SLE = systemic lupus erythematosus, SS = Sjögren’s syndrome.

In October 2020, a long-acting protocol was used in the luteal phase to superinduce ovulation, and conventional IVF was conducted as the semen of the husband was normal. Three days after oocyte retrieval, 2 day 3 cleavage-stage high-quality embryos were transferred in this fresh cycle (embryo transfer was performed in the same cycle as oocyte retrieval). Based on the previous 2 failed IUI attempts with only HCQ 0.1g BID oral treatment, another immunosuppressive agent was added in this IVF cycle, combining prednisone 5 mg QD orally with HCQ 0.1 g BID orally. However, no clinical pregnancy was established. Three blastocysts (2 high-quality blastocysts and 1 fair-quality blastocyst) were cryopreserved for future frozen–thawed embryo transfer.

In December 2020, the patient had a spontaneous pregnancy, but the fetus ceased to develop at 7 weeks of gestation and the pregnancy terminated with an abortion. The dose of immunosuppressive agent might be insufficient to resist the high levels of immunotoxicity, leading to spontaneous miscarriage. Therefore, higher doses of immunosuppressive agents were administered, with a dose of prednisone 5mg/d QD (5 mg QD) and HCQ 0.4 g/d (0.2 g BID). Unexpectedly, the implantation both failed in the following 2 cycles of frozen embryo transfer (the first cycle in April 2021 and the second cycle in January 2022).

At this point, all the frozen embryos available for transplantation had been used, so the patient underwent the second cycle of conventional IVF in March 2022. Considering that too many oocytes (30 oocytes) were retrieved and the possibility of ovarian hyperstimulation syndrome, the patient was advised not to transfer any embryos during the fresh cycle and all embryos obtained were frozen.

The effectiveness of the regimen prednisone 5mg QD combined with HCQ 0.2 g BID was questioned due to the 2 consecutive failures of frozen embryo transfer despite optimization of the immune treatment regimen (Table 2). In July 2022, a high-quality frozen blastocyst embryo was transplanted and another more potent immunosuppressive agent cyclosporine A (CsA) was added (Table 2). The transplantation resulted in clinical pregnancy followed by biochemical pregnancy with human chorionic gonadotropin (hCG) positive in blood. Notably, the dose of CsA was halved when a serum hCG increase of more than 66% over 48 hours (the hCG ratio > 1.66) was achieved; CsA administration was discontinued when the gestational sac and fetal heart buds were detected by ultrasound. After confirming the clinical pregnancy, regular prenatal examinations were conducted throughout the pregnancy. The fetus was safely delivered by elective cesarean section (Table 3). At the 1-year follow-up, both the pregnant woman and the fetus were healthy. Moreover, a follow-up examination at 1 year of age confirmed the child’s physical and mental health with normal height, weight, intelligence, and neurologic development. A written informed consent was obtained from the patient for the publication of this case report, and the study was approved by the Luoyang Maternal and Child Hospital’s Medical Ethics Committee. All procedures were performed in accordance with the Declaration of Helsinki.

**Table 2:** History of assisted reproduction.

Number	Method	Date	Therapeutic regimen of IM	Embryo quality for ET	Outcome
1	IUI	08/2020	HCQ 0.1 g bid	–	No pregnancy
2	IUI	09/2020	HCQ 0.1g bid	–	No pregnancy
3	IVF	10/2020	P 5 mg qd HCQ 0.1 g bid	High	No pregnancy
	Spontaneous pregnancy	12/2020	Same as before	–	Fetal loss at the 10th wk of gestation
4	FET	04/2021	P 5 mg qd HCQ 0.2 g bid	Fair	No pregnancy
5	FET	01/2022	same as before	High	No pregnancy
6	IVF	03/2022	–	–	–
7	FET	07/2022	CsA 50 mg bid	High	Delivery

CsA = cyclosporine A, ET = embryo transfer, FET = frozen embryo transfer, HCQ = hydroxychloroquine, IM = immunosuppressive agents, IUI = intrauterine insemination, IVF = in vitro fertilization, P = prednisone.

**Table 3:** Outcomes and follow-up course of the patient after the final embryo transplantation.

Follow‐up schedule	Stage of pregnancy	Outcome	Adjustment of CsA dosage
First trimester	＜5 wk	Pregnancy confirmation by serum β-hCG	
	FET 10 d	hCG: 170.51 mIU/mL	Dosage maintained at 50 mg bid
	FET 12 d	hCG: 809.17 mIU/mL	
	FET 17 d	hCG: 3368.06 mIU/mL	Dosage halved to 25 mg bid
	7 wk	Pregnancy confirmation by ultrasound	Discontinued
	7 to 13^+6^ wk	Maternal-fetal healthy	
Second trimester	14 to 27^+6^ wk	Maternal-fetal healthy	
Third trimester	28 to 39 wk	Maternal-fetal healthy	
	39^+2^ wk	Full-term delivery	

CsA = cyclosporine A, FET = frozen embryo transfer, hCG = human chorionic gonadotropin.

## 3. Discussion

Infertility is a common clinical disease. The incidence of infertility is constantly rising due to factors such as delayed childbearing age, environmental pollution, and life pressure. The incidence of infertility is about 10% to 12%,^[24]^ with immune infertility accounting for 10% to 30%.^[25]^ The term “immune infertility” was first reported by Shulman et al in 1978^[26]^; however, the concept of immune infertility remains controversial in modern medicine. Most doctors refer to immune infertility as the presence of serum autoimmune antibodies impairing fertility in the absence of female ovulation disorders, reproductive system dysfunction, abnormal semen, pathogenic factors, and other factors.

Sjögren’s syndrome is a chronic inflammatory autoimmune disease that can be further subdivided into primary Sjögren syndrome (pSS) and secondary SS (sSS). The latter refers to cases in which it coexists with another connective tissue disorder, such as rheumatoid arthritis or systemic lupus erythematosus.^[27–30]^ Most patients with pSS have an occult onset with varying clinical manifestations. Dry eyes and mouth, fatigue, and joint pain are the typical clinical symptoms. In this study, the patient presented with typical pathological features and blood antibodies of SS, but she did not feel significant clinical symptoms of dry eyes and mouth. At this time, an interesting interrogative were raised – are clinical symptoms of this patient more insidious and difficult to find out? To address this, a detailed history focused on lifestyle was surveyed and it revealed that the patient did not regularly drink water due to her hectic job. Hence, the patient was not asymptomatic, but rather may develop tolerance in the body’s nervous system, producing no sensation of thirst and dry eye. Therefore, the medical history of the patient should be carefully evaluated to offer support and to make a definite diagnosis.

Notably, pSS is a global disease that is mostly seen in women (female-to-male ratio 8–9: 1),^[31–33]^ with a prevalence rate of approximately 0.1 to 4.8%.^[32,34,35]^ In recent years, a growing number of clinical studies have shown that SS is associated with a higher risk of adverse maternal and fetal outcomes.^[36,37]^ The most severe outcome is congenital heart block.^[34,35]^ It is related to the transplacental passage of maternal anti-SSA and anti-SSB antibodies, which may mediate tissue damage of the atrioventricular node.^[35]^ It occurs in approximately 1% to 2% of offspring of a mother with anti-SSA antibodies.^[31,33,35]^ Other adverse outcomes associated with SS include an increased risk of miscarriage and fetal loss,^[34,36,38–40]^ a higher incidence of preterm delivery,^[32,36,39]^ and fetal growth restriction.^[32]^ However, research investigating the relationship between SS and infertility is relatively scarce. The studies mentioned above also focused on whether SS could lead to infertility; however, the conclusions remain controversial. In this couple, the causes of infertility were comprehensively screened, and only immune factors were detected in the female patient. Therefore, SS was likely the leading cause of infertility. Notably, a potential reproductive barrier caused by severe acute respiratory syndrome-coronavirus 2 (SARS-CoV-2) cannot be completely ruled out.^[41,42]^

The maternal immune system is highly dynamic and regulated during pregnancy,^[43]^ which is similar to a successful semi-allogeneic transplantation process. Due to the interaction between the mother and the fetus, the maternal immune function can protect the mother from immune rejection of the intrauterine embryo transplant, maintaining pregnancy. In contrast, impaired maternal immune function may result in repeated pregnancy loss. Therefore, normal maternal immune function and maternal-fetal tolerance are the key foundations of pregnancy. Up to date, multiple studies have reported that the immunosuppressive agent treatment could improve pregnancy outcomes of patients with positive autoimmune antibodies and potentially abnormal immune function.^[44–47]^

Inevitably, using an immunosuppressive agent also raises concerns such as the optimal dose, its adjustment, duration, and side effects. In terms of side effects, previous studies reported that the use of immunosuppressive agents such as HCQ,^[48–51]^ prednisone,^[52]^ and CsA^[53,54]^ has not been associated with adverse pregnancy outcomes. However, it should be noted that optimal dose, dose adjustment and treatment duration have rarely been described in detail. Notably, this case report presented the continuous optimization process of immunosuppressive agent treatment, and also the dosage, frequency, and duration of each immunosuppressive agent (Tables 2 and 3). This report will undoubtedly provide clinicians with valuable diagnostic and therapeutic experience.

In conclusion, this is the first report of a successful pregnancy and delivery following immune treatment and ART regimen optimization in an infertility female due to SS. So a detailed treatment process of immunosuppressive agent and assisted reproductive technology were presented in this report. In summary, this case would provide novel insights into the immunosuppressive treatment of infertile patients with SS or other autoimmune diseases, especially in patients with poor response to glucocorticoid and HCQ.

## Author contributions

**Conceptualization:** Kaiyue Hu.

**Data curation:** Shuxia Ma, Ruirui Li, Ying Zhou.

**Funding acquisition:** Kaiyue Hu.

**Methodology:** Kaiyue Hu.

**Resources:** Kaiyue Hu.

**Supervision:** Kaiyue Hu.

**Writing – original draft:** Kaiyue Hu.

**Writing – review & editing:** Shuxia Ma, Ruirui Li.
